# Sex differences in cardiovascular risk, lifestyle, and psychological factors in patients with type 2 diabetes: the Fukuoka Diabetes Registry

**DOI:** 10.1186/s13293-023-00517-8

**Published:** 2023-05-22

**Authors:** Toshiaki Ohkuma, Masanori Iwase, Hiroki Fujii, Takanari Kitazono

**Affiliations:** 1 Department of Medicine and Clinical Science, Graduate School of Medical Sciences, Kyushu University, Maidashi 3-1-1, Higashi-Ku, Fukuoka, 812-8582 Japan; 2 Diabetes Center, Hakujyuji Hospital, Fukuoka, Japan; 3 Division of Internal Medicine, Fukuoka Dental College, Fukuoka, Japan

**Keywords:** Cardiovascular risk factor, Diabetes, Lifestyle, Psychosocial factor, Sex difference

## Abstract

**Background:**

The excess risk of cardiovascular diseases associated with diabetes is greater in women than in men. The present study aimed to examine sex differences in the control of cardiovascular risk factors, as well as lifestyle and psychological factors, in patients with type 2 diabetes.

**Methods:**

A total of 4923 Japanese patients with type 2 diabetes were included in this cross-sectional study. Female/male differences in cardiovascular risk factor levels, and corresponding odds ratios for achieving recommended ranges for preventing cardiovascular diseases and having unhealthy lifestyle and psychological factors were computed by linear and logistic regression models.

**Results:**

Women were less likely than men to achieve recommended ranges for glycated hemoglobin, low-density lipoprotein cholesterol, non-high-density lipoprotein cholesterol, and obesity-related anthropometric indices such as body mass index and waist circumference, but were more likely than men to be on target for high-density lipoprotein cholesterol and triglycerides. Women were also more likely than men to have an unhealthy lifestyle and psychological factors, including less dietary fiber intake, less leisure-time physical activity, shorter sleep duration, more constipation, and more depressive symptoms. Similar findings were observed when the participants were subgrouped by age (< 65 and ≥ 65 years) and past history of cardiovascular disease.

**Conclusions:**

We observed significant sex differences for a range of cardiovascular risk factors, as well as lifestyle and psychological factors, suggesting the importance of adopting a sex-specific approach for the daily clinical management of diabetes.

**Supplementary Information:**

The online version contains supplementary material available at 10.1186/s13293-023-00517-8.

## Plain Language Summary

Diabetes increases the risk of cardiovascular diseases, and growing evidence suggests that the risk increases more in women than men. Differences between the sexes in terms of the control of risk factors have been proposed to explain this association. Although ethnic and regional differences in the management of cardiovascular risk factors have been reported, most evidence has come from Western countries, and evidence from Asia is limited. Given the differences in health care systems, as well as cultural and sociological backgrounds, it is important to clarify the sex differences in the management of cardiovascular risk factors, lifestyle, and psychological factors in order to incorporate appropriate sex-specific approaches into public health policies.

The present study comprehensively assessed sex differences in a wide range of cardiovascular risk factors, as well as lifestyle and psychological factors in Japanese patients with type 2 diabetes. The results showed that women were less likely than men to achieve recommended ranges for glycemic control, low-density lipoprotein-cholesterol and non-high-density lipoprotein-cholesterol, as well as obesity-related anthropometric indices, but were more likely to be on target for high-density lipoprotein-cholesterol and triglycerides. In addition, women were more likely to have unhealthy lifestyle and psychological factors, such as less dietary fiber intake, less physical activity, shorter sleep duration, and more constipation, and depressive symptoms. These results suggest the need for a comprehensive and sex-specific approach for the management of cardiovascular risk factors, as well as lifestyle and psychological factors, to reduce the risk of cardiovascular diseases in patients with diabetes.

**Supplementary Information:**

The online version contains supplementary material available at 10.1186/s13293-023-00517-8.

## Highlights

- Evidence on sex differences in the control of cardiovascular risk factors, as well as lifestyle and psychological factors, in patients with type 2 diabetes is limited, especially in Asia, where the health care systems and cultural and sociological backgrounds differ from Western countries.
- The present study of Japanese patients with type 2 diabetes showed that women were less likely than men to achieve recommended ranges for HbA_1c_, LDL-C, non-HDL-C, and obesity-related anthropometric indices, but were more likely to be on target for HDL-C and triglycerides.
- Women were more likely than men to have unhealthy lifestyle and psychological factors, including less dietary fiber intake, less physical activity, shorter sleep duration, and more constipation and depressive symptoms.
- These findings suggest the importance of comprehensive and sex-specific approaches for managing cardiovascular risk factors, as well as lifestyle and psychological factors, in the daily clinical management of diabetes.

**Supplementary Information:**

The online version contains supplementary material available at 10.1186/s13293-023-00517-8.

## Background

Cardiovascular diseases (CVDs) are the leading cause of morbidity and mortality in patients with diabetes [1]. Comprehensive assessment and treatment of CVD risk factors, such as obesity/overweight, hypertension, dyslipidemia, and smoking, are recommended to prevent their occurrence [1]. Recent growing evidence has suggested that diabetes is a stronger risk factor for CVDs, such as coronary heart disease [2], stroke [3], and heart failure [4], in women than in men. A similar sex difference in the hazardous impact of diabetes was also observed for non-vascular diseases such as cancer [5]. These findings indicate that the female advantage with respect to the risks of these diseases lessens in patients with diabetes; i.e., women with diabetes are likely to catch up to some extent if they have diabetes [6].

The mechanisms responsible for the greater diabetes-related consequences in women compared with men are uncertain. Sex disparities in diabetes control may be involved [3–5], as well as sex differences in the control of other cardiovascular risk factors [2–4], suggesting the importance of sex-specific management of diabetes and cardiovascular risk factors. Ethnic differences in the management of cardiovascular risk factors have also been reported [7], but the available evidence on this topic is limited in Asia, especially in Japan. Furthermore, although positive health behaviors and maintaining psychological well-being are fundamental aspects of diabetes management [1], sex differences in these factors remain unclear. Differences in health care systems, as well as cultural and sociological backgrounds, across countries, highlight the need to clarify sex differences in the management of cardiovascular risk factors, as well as lifestyle and psychological factors, in order to incorporate sex-specific approaches into public health policies.

The present study thus aimed to examine sex differences in the control of cardiovascular risk factors, as well as a range of lifestyle and psychological factors, within a cohort of Japanese patients with type 2 diabetes.

## Research design and methods

### Study design and population

The Fukuoka Diabetes Registry is a multicenter prospective cohort study designed to examine the influence of contemporary treatments on the prognosis of patients with diabetes who regularly attend teaching hospitals or diabetes clinics certified by the Japan Diabetes Society in Fukuoka Prefecture, Japan [8]. A total of 5131 outpatients with diabetes aged ≥ 20 years were recruited between April 2008 and October 2010. The exclusion criteria were as follows: (1) drug-induced diabetes or receiving steroid treatment; (2) renal replacement therapy; (3) serious diseases other than diabetes, such as advanced malignancies or decompensated liver cirrhosis; and (4) patients unable to regularly visit a hospital or clinic. After excluding 208 patients with type 1 diabetes (negative serum C-peptide under insulin treatment), this cross-sectional study included a total of 4923 patients with type 2 diabetes.

### Clinical evaluation and laboratory measurements

Participants completed a self-administered questionnaire covering their medical history, medication use, dietary habits, alcohol consumption, smoking, sleep duration, leisure-time physical activity (LTPA), defecation frequency, laxative use, and depressive symptoms. Dietary habits were evaluated using a brief-type self-administered diet history questionnaire (Gender Medical Research Inc., Tokyo, Japan) based on the frequency of consumption of 58 items, which has been validated for ranking energy-adjusted dietary fiber intake in Japanese adults [9]. LTPA was evaluated using a self-administered questionnaire, and metabolic equivalent hours per week (MET·h/w) were calculated [10]. Depressive symptoms were assessed using the Center for Epidemiologic Studies Depression Scale (CES-D) [11]. Blood was collected by venipuncture. Glycated hemoglobin (HbA_1c_) was determined using high-performance liquid chromatography (Tosoh, Tokyo, Japan), and total cholesterol, high-density lipoprotein cholesterol (HDL-C), low-density lipoprotein cholesterol (LDL-C), and triglyceride levels were determined by enzymatic methods. Body mass index (BMI) was calculated as weight (kg) divided by height^2^ (m). Waist circumference was measured at the umbilical level in the standing position. Blood pressure (BP) was measured with the participant in the sitting position.

### Study outcomes

The study outcomes were levels of cardiovascular risk factors, including HbA_1c_, BP (systolic, diastolic, and pulse pressure), lipids (HDL-C, LDL-C, non-HDL-C, and triglycerides), anthropometric indices (BMI and waist circumference), and lifestyle and psychological factors (dietary fiber intake, LTPA, sleep duration, defecation frequency, and CES-D score). The proportions of participants who achieved recommended ranges for cardiovascular risk factors and lifestyle and psychological factors were also assessed. The recommended ranges were defined as HbA_1c_ < 7%, BP < 130/80 mmHg, LDL-C < 2.59 mmol/L and < 3.11 mmol/L (in participants with and without a history of coronary heart disease, respectively), non-HDL-C < 3.37 mmol/L and < 3.89 mmol/L (in participants with and without a history of coronary heart disease, respectively), HDL-C ≥ 1.03 mmol/L, and fasting triglycerides < 1.68 mmol/L [12]. BP < 140/90 mmHg was also assessed as BP control. For anthropometric indices, obesity was defined as BMI ≥ 25 kg/m^2^ [13] and abdominal obesity as waist circumference ≥ 80 cm in women and ≥ 90 cm in men, based on the definition of abdominal obesity for Asians [14]. The proportions of participants who received prescriptions of glucose-lowering, anti-hypertensive, and anti-hyperlipidemic medications were also evaluated. Anti-hyperlipidemic medications included statins, fibrates, ezetimibe, bile acid sequestrant, probcol, niacin, and icosapent ethyl. Smoking habit was classified as either current or not. Participants were categorized as consuming less dietary fiber if their consumption was < 20 g/day, according to the Japanese Clinical Practice Guideline for Diabetes [12]. Participants with ≥ 6.6 MET·h/w were categorized as physically active [15, 16]. A sleep duration < 6 h/day was categorized as a short sleep duration, as frequently defined in epidemiological studies, and based on results showing increased cardiometabolic risk in patients with type 2 diabetes [17]. Constipation was defined as defecation frequency < 3 times/week and/or taking laxative medication, in accord with the major symptoms of constipation in the Rome IV criteria [18]. Participants with a CES-D score ≥ 16/60 points were considered to have depressive symptoms.

### Statistical analysis

Participant characteristics according to sex are presented as mean (standard deviation) for continuous variables, and number (percentage) for categorical variables. Differences in characteristics between women and men were analyzed by unpaired *t*-test or χ^2^ test, as appropriate. Triglyceride levels were presented as median (interquartile interval) and log-transformed for statistical analyses because of a skewed distribution.

Age-adjusted mean values for cardiovascular risk factors were analyzed by analysis of covariance, and female/male differences were calculated using linear regression models. Female/male odds ratios (ORs) for achieving recommended ranges or having unhealthy lifestyle and psychological factors were estimated by logistic regression models with adjustment for age. Subgroup analyses were carried out according to baseline age (< 65 and ≥ 65 years) and history of CVD (coronary heart disease or stroke). Heterogeneity across subgroups was estimated by adding an interaction term to the relevant model. Models were adjusted for age throughout, except for subgroup analyses by age. Statistical analyses were conducted using SAS version 9.4 (SAS Institute, Cary NC, USA). A two-sided P-value < 0.05 was considered to be statistically significant.

## Results

The clinical characteristics of study participants according to sex are shown in Table 1. Among the participants included in the present analyses, 43.3% (n = 2133) were women and the mean ages of the women and men were 66 and 65 years, respectively. Women were more likely than men to have a shorter duration of diabetes, and were less likely to have a past history of CVD. The proportion of current alcohol drinkers was lower among women than men, but uses of insulin, anti-hypertensive agents, anti-hyperlipidemic agents, statins, and laxatives were higher among women compared with men. Most of the women (92.3%) were postmenopausal.

**Table 1:** Baseline characteristics according to sex

Variable	Women	Men	P value
	(n = 2133)	(n = 2790)	
Age (years)	66 (10)	65 (10)	0.007
Duration of diabetes mellitus (years)	14.3 (9.8)	16.5 (10.9)	< 0.001
History of cardiovascular disease (%)	17.4% (370)	25.0% (697)	< 0.001
History of coronary heart disease (% )	10.8% (230)	16.1% (449)	< 0.001
History of stroke (%)	8.5% (181)	11.8% (328)	< 0.001
Current alcohol drinking (%)	16.5% (351)	56.5% (1,575)	< 0.001
Total energy intake (kcal/day)	1,508 (412)	1,826 (506)	< 0.001
Oral hypoglycemic agent use (%)	63.5% (1,355)	63.2% (1,762)	0.79
Insulin use (%)	30.8% (656)	27.4% (763)	0.01
Anti-hypertensive agent use (%)	55.7% (1,188)	52.7% (1,469)	0.04
Anti-hyperlipidemic agent use (%)	58.4% (1,246)	41.1% (1,147)	< 0.001
Statins use (%)	53.7% (1,146)	35.5% (991)	< 0.001
Laxative use (%)	26.2% (558)	15.9% (443)	< 0.001
Menopause (%)	92.3% (1,968)		

Values shown as mean (SD) for continuous variables, and as percentage (number) for categorical variables

Percentage of postmenopausal participants was calculated among women

**Fig. 1:**
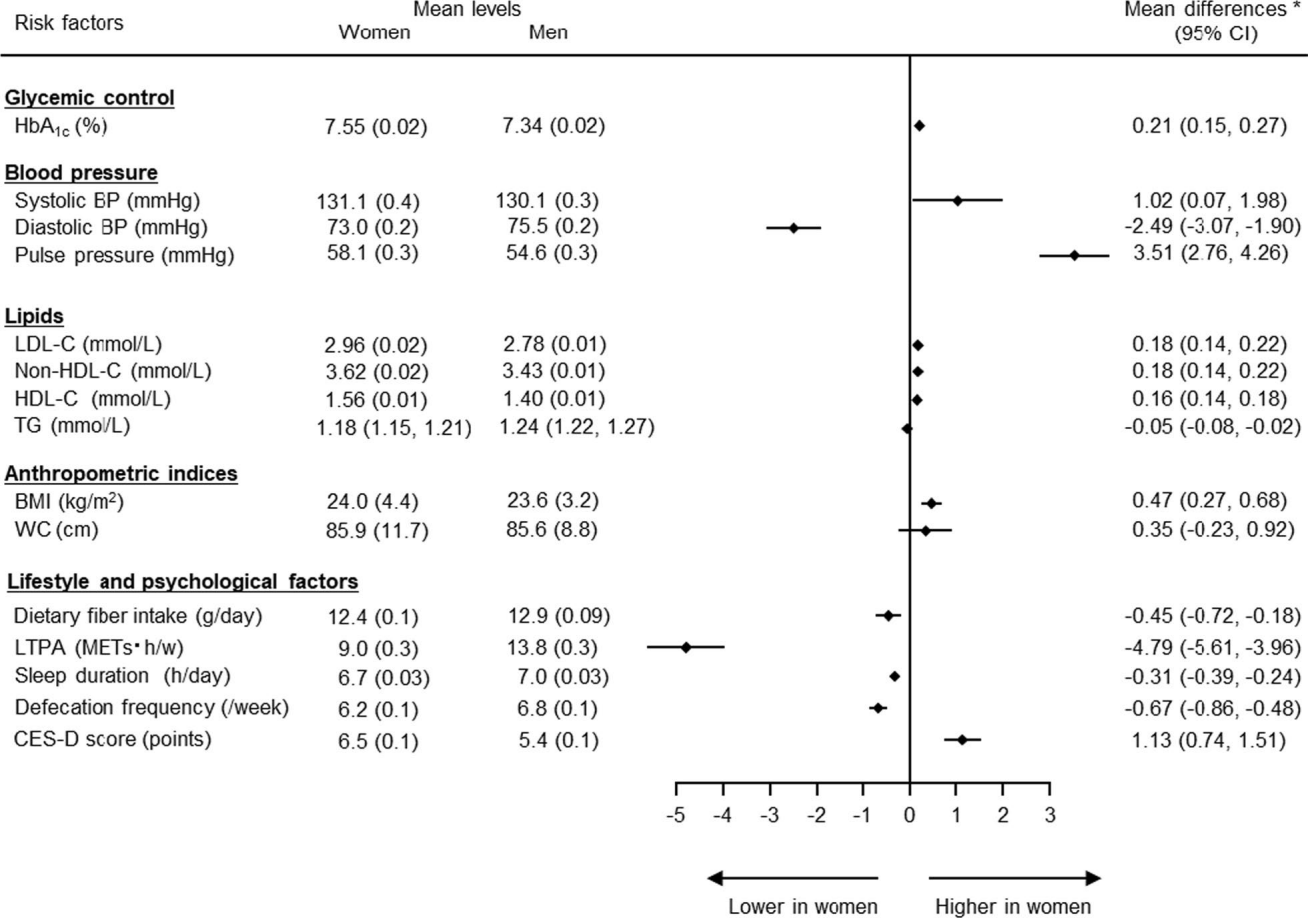
Cardiovascular risk factor levels in men and women. Mean values and female/male differences adjusted for age. Mean levels presented as mean (standard error). Triglycerides presented as geometric mean (95% CI), and differences calculated after log-transformation among participants with available data for fasting values (n: women = 1929, men = 2512). Six participants with missing values for defecation frequency were excluded from the analysis of constipation. *BMI* body mass index, *BP* blood pressure, *CES-D* Center for Epidemiologic Studies Depression, *CI* confidence interval, *HDL-C* high-density lipoprotein cholesterol, *LDL-C* low-density lipoprotein cholesterol, *LTPA* leisure-time physical activity, *TG* triglycerides

The sex-specific mean values and differences in cardiovascular risk factors and lifestyle and psychological factors, after adjusting for age, are shown in Fig. 1. HbA_1c_ was significantly higher in women than men by 0.21%. Systolic BP and pulse pressure were also significantly higher in women than men by 1.02 mmHg and 3.51 mmHg, respectively, while diastolic BP was higher in men than women by 2.49 mmHg. The corresponding female/male differences in LDL-C, non-HDL-C, HDL-C, and BMI were 0.18 mmol/L, 0.18 mmol/L, 0.16 mmol/L, and 0.47 kg/m^2^, respectively Triglycerides and waist circumference levels were similar in women and men. In terms of lifestyle and psychological factors, women had lower levels of dietary fiber intake, LTPA, sleep duration, and defecation frequency, but higher CES-D scores compared with men.

**Fig. 2:**
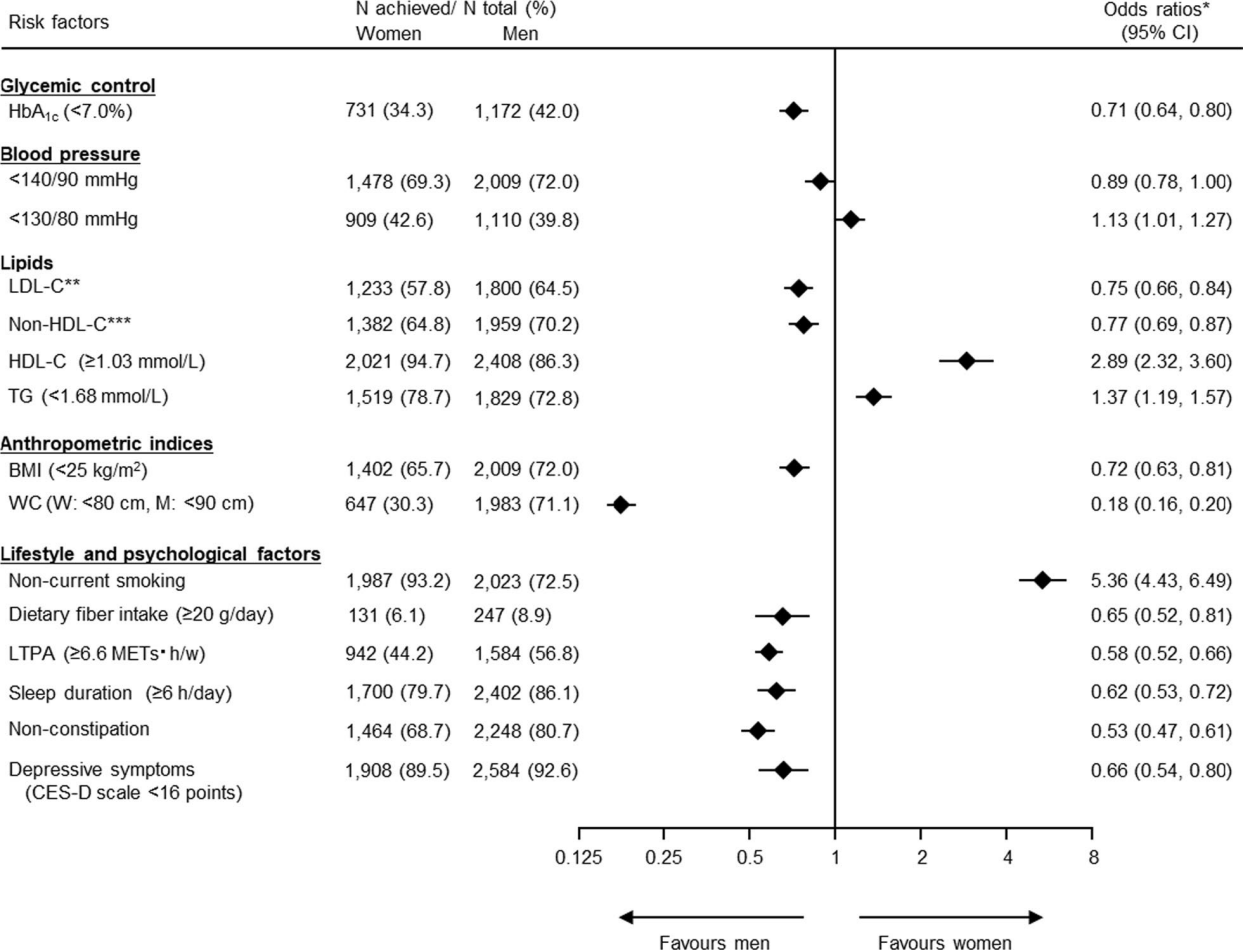
Achievement of recommended ranges for cardiovascular risk factors in men and women. *Female/male odds ratios adjusted for age. **LDL-C < 2.59 mmol/L in participants with a history of coronary heart disease, < 3.11 mmol/L in those without a history of coronary heart disease. ***Non-HDL-C < 3.37 mmol/L in participants with a history of coronary heart disease, < 3.89 mmol/L in those without a history of coronary heart disease. Triglycerides evaluated among participants with available data for fasting values (n: women = 1929, men = 2512). Six participants with missing values for defecation frequency were excluded from the analysis of constipation. *BMI* body mass index, *BP* blood pressure, *CES-D* Center for Epidemiologic Studies Depression, *CI* confidence interval, *HDL-C* high-density lipoprotein cholesterol, *LDL-C* low-density lipoprotein cholesterol, *LTPA* leisure-time physical activity, *TG* triglycerides

We also assessed sex differences in the proportions of participants who achieved recommended ranges for cardiovascular risk factors, and had unhealthy lifestyle and psychological factors (Fig. 2). Women were less likely to reach recommended range for HbA_1c_ levels than men (34.3% and 42.0%, respectively), with an age-adjusted female/male OR for achieving HbA_1c_ < 7.0% of 0.71 (95% confidence interval [CI] 0.64–0.80). Regarding BP control, 69.3% of women and 72.0% of men achieved BP < 140/90 mmHg; however, women were more likely than men to achieve the more stringent BP level of < 130/80 mmHg (42.6% and 39.8%, respectively), though the sex differences were relatively small in both cases (< 3%). The corresponding female/male ORs for achieving BPs < 140/90 mmHg and < 130/80 mmHg were 0.89 (0.78–1.00) and 1.13 (1.01–1.27), respectively. Regarding lipid control, women were less likely than men to achieve recommended ranges for LDL-C (age-adjusted OR: 0.75 [95% CI 0.66–0.84]) and non-HDL-C (0.77 [0.69–0.87]), and more likely to achieve recommended ranges for HDL-C (2.89 [2.32–3.60]) and triglyceride (1.37 [1.19–1.57]). Additional adjustments for duration of diabetes, BMI, and medication use were also conducted (Additional file 1: Fig. S1). Multiple-adjusted female/male OR for achieving HbA_1c_ < 7.0% was 0.69 (0.61–0.78). Further adjustment for statins use did not materially change the result (0.72 [0.63–0.82]). Similarly, the direction of the findings did not change significantly for BP and lipids control. In terms of anthropometric indices, women were less likely than men to reach recommended ranges, with female/male ORs of 0.72 (0.63–0.81) for obesity and 0.18 (0.16–0.20) for abdominal obesity.

In terms of lifestyle and psychological factors, women were generally more likely to have unhealthy factors than men, with the exception of smoking. Women consumed less dietary fiber (OR 0.65 [0.52–0.81]), had less exercise (OR 0.58 [0.52–0.66]), shorter sleep duration (OR 0.62 [0.53–0.72]), and more constipation (OR 0.53 [0.47–0.61]) and depressive symptoms (OR 0.66 [0.54–0.80]) than men. On the other hand, women were less likely than men to be current smokers (OR 5.36 [4.43–6.49]). Sensitivity analyses excluding premenopausal women did not significantly alter the results (Additional file 1: Fig. S2). In addition, the above sex differences did not differ between subgroups defined by age (< 65 and ≥ 65 years) (Additional file 1: Table S1). Although significant heterogeneity was found for BMI, waist circumference, dietary fiber intake, and LTPA, the directions of the results were similar across subgroups, with the exception of dietary fiber intake. Subgroup analyses of sex differences according to a previous history of CVD also showed no significant heterogeneity, except for LTPA and constipation (Additional file 1: Table S2).

## Discussion

The present study showed the existence of sex differences in terms of the control of cardiovascular risk factors, as well as a range of lifestyle and psychological factors, in Japanese patients with type 2 diabetes. Women were less likely than men to achieve recommended ranges for HbA_1c_, LDL-C, non-HDL-C, and obesity-related anthropometric indices, such as BMI and waist circumference, but were more likely to be on target for HDL-C and triglycerides. Women were more likely than men to have unhealthy lifestyle and psychological factors, such as less dietary fiber intake, less LTPA, shorter sleep duration, and more constipation and depressive symptoms. The observed sex differences were broadly consistent between subgroups defined by age and history of CVD. These findings highlight the importance of sex-specific management strategies for patients with type 2 diabetes.

Previous large-scale meta-analyses demonstrated greater excess risks of diabetes complications in women than men [2–5]. For instance, women with diabetes had a 44% greater excess risk of incident coronary heart disease compared with men with diabetes, with pooled relative risks of 2.82 (95% CI 2.35–3.38) in women and 2.16 (1.82–2.56) in men [2]. The corresponding excess risk of stroke associated with diabetes was also 27% greater in women than men [3]. These excess risks of adverse consequences associated with diabetes have been attributed to sex differences in the management and treatment of cardiovascular risk factors [19], including glycemia, BP, and lipid control. Regarding sex differences in glycemic control, most studies showed that women had a higher likelihood of poorer control than men [20–27], although several studies found no differences between women and men [28–31] or worse control in men [32, 33]. Differences in BP control between women and men varied across studies, with different studies showing worse control in women [20, 25, 31–34] or in men [23, 29], or no sex differences [21, 28, 30]. Regarding lipid control, women consistently showed worse control than men for LDL-C [21–27, 29–36] and non-HDL-C [25, 26, 35], with some exceptions [28], while sex differences in HDL-C and triglycerides yielded mixed results [21, 22, 24–29, 34, 35]. Obesity-related indices consistently showed a worse profile in women compared with men [20–23, 25–29, 32–34, 36].

These sex differences in cardiovascular risk factors have mainly been reported in Western countries and few studies have examined these issues in Asia, especially in Japan, where the genetic and environmental backgrounds, incidence of CVDs, and access to health care resources differ. A previous study conducted among Japanese patients with diabetes showed higher BMI, HbA_1c_, systolic BP, and LDL-C and HDL-C levels, and lower triglyceride levels in women than in men [26], although the results were unadjusted crude values. In addition, sex differences in the proportions of patients who achieved recommended ranges were not evaluated. The present study showed that women were less frequently on target for glycemic control, LDL-C, non-HDL-C, and obesity-related anthropometric indices, but more likely to have HDL-C and triglyceride levels under control. Sex differences in BP control varied depending on the cut-off values used. These sex differences were broadly consistent with those observed in Western countries, thus extending them to Japanese patients.

As noted above, previous studies have assessed sex differences in cardiovascular risk factors; however, evidence for sex differences in lifestyle and psychological factors, which are also associated with the risk of CVD, is relatively limited. Regarding physical activity, women with diabetes were reported to be physically inactive compared with their male counterparts [20, 26, 33, 37, 38]. A systematic review and meta-analysis demonstrated that adult female patients with diabetes were less likely than male patients to meet physical activity guidelines and performed less moderate-to-vigorous physical activity [38]. Insufficient sleep quantity or short sleep duration, as important components of insomnia, were also associated with deteriorations in a range of cardiovascular risk factors, including glycemic control, obesity, BP, and lipid levels [39, 40]. Although data for patients with diabetes is scarce, a previous meta-analyses reported an increased risk of insomnia in women than in men in the general population [41]. The prevalence of depression has also been reported to be higher in women than in men among patients with diabetes [20, 28, 33]. A systematic review and meta-analysis of population-based studies also showed a higher prevalence of constipation in women compared with men [42], and a female dominance in the prevalence of constipation was also observed among patients with diabetes [43]. In contrast, consistently more men than women reported a smoking habit among patients with diabetes [22, 23, 27, 30, 32–34, 36]. Findings regarding sex differences in dietary fiber consumption among patients with diabetes have been limited and inconsistent. A study conducted in the USA showed that women with diabetes were more likely to report high fruit and vegetable consumption, as a major source of dietary fiber, compared with men with diabetes [33], while a study from the National Health and Nutrition Examination Survey found no difference between the sexes [37], and another study among Japanese patients with diabetes also reported no significant sex difference in the prevalence of meeting the recommended dietary fiber intake (≥ 20 g/day) [26].

In the present study, women were more likely than men to have unhealthy lifestyle and psychological factors, such as consuming less dietary fiber, being physically inactive, sleeping less, and having constipation and depressive symptoms, whereas men were more likely to smoke. These findings were broadly consistent with the results of previous studies mainly conducted in Western countries, and provide additional evidence relevant to Japanese patients, who have different cultural background and lifestyle factors. In addition, most previous studies on this topic evaluated lifestyle and psychological factors individually, and only a few studies have assessed multiple factors simultaneously [33]. Notably, to the best of our knowledge, the current study provides the first comprehensive assessment of sex differences in a wide range of lifestyle and psychological factors, in addition to cardiovascular risk factors, within the same cohort of patients with diabetes. Taken together with the fact that several conventional risk factors were more strongly associated with the risk of CVD in women than men [6, 44], the current findings provide valuable evidence to reinforce the importance of the sex-specific management of risk factors.

There are several possible explanations for the observed sex differences in cardiovascular risk factors, including biological differences between women and men. Differences in obesity-related anthropometric indices, including greater BMI or waist circumference, may explain the worse risk factor profile in women than in men [19]. In addition, sex differences in pharmacological responses to drugs, including their absorption, distribution, metabolism, and excretion, may also be responsible for the sex-related association [45]. Notably, systolic BP levels were higher in women compared with men, despite the fact that significantly more women received anti-hypertensive agents than men. In addition to biological differences, poorer adherence to medication in women than men [46] may also help to account for the sex differences, with women having been shown to be likely to be less adherent to medication [35, 46]. This hypothesis is supported by the fact that similar proportions of men and women used oral diabetic medications in the present study, and more women than men used insulin, but women were still less likely than men to achieve recommended ranges for glycemic levels. However, the observed sex differences remained unchanged after adjustment for medication use. For example, women were less likely than men to achieve HbA_1c_ < 7.0% even after adjustment for oral hypoglycemic agent, insulin, and statins which was associated with hyperglycemia [47], suggesting that other mechanisms may be involved. Female-specific reproductive factors, such as age at menarche, age at menopause, and childbearing history, may also play a role [21] and have previously been associated with the future risk of CVD [48]. Another study showed that earlier age at menarche was associated with obesity and poor glycemic control in patients with type 2 diabetes [49]. Menopause transition and its incidental hormonal changes have also been associated with unfavorable changes in lipids, accumulation of abdominal adiposity, and increased blood glucose [50], and may contribute to the observed sex differences. Sex-related differences in lifestyle behaviors and psychological factors could also contribute to the sex difference in cardiovascular risk factor control. Unhealthy lifestyle and psychological factors, such as consuming less dietary fiber, short sleep duration, decreased physical activity, constipation, and depressive symptoms were more common in women than men in this study. Sex differences in knowledge, awareness, and perception of the diseases [51, 52], as well as sex differences in cardiovascular risk factor levels at treatment initiation [21], may also contribute to the observed difference. The deterioration in cardiovascular risk factor levels in participants with diabetes compared with those without diabetes was shown to be greater in women than men [3], suggesting the need for a more intensive approach to obtain the same treatment effect in women compared with men.

The strengths of the current study included the collection of blood samples with standardized measurement methods, which reduced possible measurement errors, and the completeness of the data, including drug prescriptions, without any missing values. In addition, this study provides the first comprehensive assessment of sex differences in a wide range of lifestyle and psychological factors, as well as conventional cardiovascular risk factors, within the same cohort. However, the study also had some limitations. First, all the participants were recruited from Japan, and the generalizability of the results to other regional and ethnic populations may therefore be limited. Second, no information on adherence to medical treatment was available. Third, single measurement of BP may have resulted in misclassification. Fourth, information on potential residual confounding factors, such as educational and economic backgrounds, was not included in the present study. Finally, the cross-sectional design of the study limited any inference of a cause-and-effect relationship.

### Perspectives and significance

Significant sex differences in cardiovascular risk factors and in lifestyle and psychological factors were identified in Japanese patients with diabetes, with women generally less likely to achieve recommended ranges for risk factors and more likely to have unhealthy lifestyle habits. These results suggest the need for a more comprehensive and sex-specific approach for the management of cardiovascular risk factors, as well as lifestyle and psychological factors, to reduce the risk of CVDs in patients with type 2 diabetes.

## Supplementary Information

**Additional file 1: Table S1.** Subgroup analyses: achievement of recommended ranges for cardiovascular risk factors in men and women according to age. **Table S2. **Subgroup analyses: achievement of recommended ranges for cardiovascular risk factors in men and women according to previous history of CVD. **Figure S1. **Sensitivity analyses: achievement of recommended ranges for cardiovascular risk factors in men and women after multiple-adjustment. **Figure S2.** Sensitivity analyses: achievement of recommended ranges for cardiovascular risk factors in men and women after excluding premenopausal participants.

## Data Availability

All supporting data are available within the article and its Additional files. The datasets used and/or analyzed during the current study are available from the corresponding author on reasonable request.
