# Supplementary material for: Sex differences in cardiovascular risk, lifestyle, and psychological factors in patients with type 2 diabetes: the Fukuoka Diabetes Registry
Source: Biol Sex Differ. 2023 May 22;14:32. doi: 10.1186/s13293-023-00517-8 (PMC10201708; doi:10.1186/s13293-023-00517-8)

**Additional files**

**Table S1. Subgroup analyses: achievement of recommended ranges for cardiovascular risk factors in men and women according to age.**

| Risk factor | Age | N achieved/N total (%) | | Odds ratio* | P for |
| --- | --- | --- | --- | --- | --- |
|  | (years) | Women | Men | (95% CI) | heterogeneity |
| **Glycemic control** |  |  |  |  |  |
| HbA_1c_ (<7.0%) | <65 | 299 (32.9) | 482 (38.7) | 0.78 (0.65, 0.93) | 0.24 |
|  | ≥65 | 432 (35.3) | 690 (44.7) | 0.67 (0.58, 0.79) |  |
| **Blood pressure** |  |  |  |  |  |
| <140/90 mmHg | <65 | 661 (72.7) | 916 (73.5) | 0.96 (0.79, 1.17) | 0.23 |
|  | ≥65 | 817 (66.8) | 1,093 (70.8) | 0.83 (0.70, 0.97) |  |
| <130/80 mmHg | <65 | 413 (45.4) | 502 (40.3) | 1.24 (1.04, 1.47) | 0.16 |
|  | ≥65 | 496 (40.5) | 608 (39.4) | 1.05 (0.90, 1.22) |  |
| **Lipids** |  |  |  |  |  |
| LDL-C** | <65 | 506 (55.7) | 766 (61.4) | 0.79 (0.66, 0.94) | 0.45 |
|  | ≥65 | 727 (59.4) | 1,034 (67.0) | 0.72 (0.62, 0.84) |  |
| Non-HDL-C*** | <65 | 571 (62.8) | 842 (67.5) | 0.81 (0.68, 0.97) | 0.51 |
|  | ≥65 | 811 (66.3) | 1,117 (72.4) | 0.75 (0.64, 0.88) |  |
| HDL-C | <65 | 867 (95.4) | 1,094 (87.7) | 2.89 (2.03, 4.11) | 0.98 |
| (≥1.03 mmol/L) | ≥65 | 1,154 (94.3) | 1,314 (85.2) | 2.87 (2.17, 3.80) |  |
| TG | <65 | 631 (75.8) | 764 (68.2) | 1.46 (1.20, 1.79) | 0.42 |
| (<1.68 mmol/L) | ≥65 | 888 (81.0) | 1,065 (76.5) | 1.31 (1.07, 1.59) |  |
| **Anthropometric indices** | |  |  |  |  |
| BMI | <65 | 549 (60.4) | 782 (62.7) | 0.91 (0.76, 1.08) | <0.001 |
| (<25 kg/m^2^) | ≥65 | 853 (69.7) | 1,227 (79.5) | 0.59 (0.50, 0.70) |  |
| WC (W: <80 cm) | <65 | 304 (33.4) | 827 (66.3) | 0.26 (0.21, 0.31) | <0.001 |
| (M: <90 cm) | ≥65 | 343 (28.0) | 1,156 (74.9) | 0.13 (0.11, 0.15) |  |
| **Lifestyle and psychological factors** | | |  |  |  |
| Non-current smoker | <65 | 814 (89.6) | 775 (62.1) | 5.22 (4.10, 6.64) | 0.84 |
|  | ≥65 | 1,173 (95.8) | 1,248 (80.9) | 5.44 (4.00, 7.40) |  |
| Dietary fiber intake | <65 | 55 (6.1) | 66 (5.3) | 1.15 (0.80, 1.67) | <0.001 |
| (≥20 g) | ≥65 | 76 (6.2) | 181 (11.7) | 0.50 (0.38, 0.66) |  |
| LTPA | <65 | 376 (41.4) | 572 (45.9) | 0.83 (0.70, 0.99) | <0.001 |
| (≥6.6 MET·h/w) | ≥65 | 566 (46.2) | 1,012 (65.6) | 0.45 (0.39, 0.53) |  |
| Sleep duration | <65 | 698 (76.8) | 1,027 (82.4) | 0.71 (0.57, 0.88) | 0.10 |
| (≥6 h) | ≥65 | 1,002 (81.9) | 1,375 (89.1) | 0.55 (0.44, 0.69) |  |
| Non-constipation | <65 | 665 (73.2) | 1,064 (85.4) | 0.47 (0.38, 0.58) | 0.17 |
|  | ≥65 | 799 (65.3) | 1,184 (76.9) | 0.57 (0.48, 0.67) |  |
| Depressive symptoms  (CES-D scale <16 points) | <65 | 790 (86.9) | 1,126 (90.3) | 0.71 (0.55, 0.93) | 0.47 |
| (CES-D scale <16 points) | ≥65 | 1,118 (91.3) | 1,458 (94.5) | 0.62 (0.46, 0.83) |  |

* Female/male odds ratios adjusted for age.

** LDL-C <2.59 mmol/L in participants with a history of coronary heart disease, <3.11 mmol/L in those without a history of coronary heart disease.

*** Non-HDL-C <3.37 mmol/L in participants with a history of coronary heart disease, <3.89 mmol/L in those without a history of coronary heart disease.

Triglycerides evaluated among participants with available data for fasting values (n: women = 1,929, men = 2,512).

Six participants with missing values in defecation frequency were excluded from the analysis of constipation.

Abbreviations: BMI, body mass index; BP, blood pressure; CES-D, Center for Epidemiologic Studies Depression; CI, confidence interval; HDL-C, high-density lipoprotein cholesterol; LDL-C, low-density lipoprotein cholesterol; LTPA, leisure-time physical activity; M, men; MET h/w, metabolic equivalent hours per week; TG, triglycerides; W, women.

**Table S2. Subgroup analyses: achievement of recommended ranges for cardiovascular risk factors in men and women according to previous history of CVD.**

| Risk factor | Hx of | N achieved/N total (%) | | Odds ratio* | P for |
| --- | --- | --- | --- | --- | --- |
|  | CVD | Women | Men | (95% CI) | heterogeneity |
| **Glycemic control** |  |  |  |  |  |
| HbA_1c_ (<7.0%) | - | 613 (34.8) | 897 (42.9)  ) | 0.70 (0.62, 0.80) | 0.99 |
|  | + | 118 (31.9) | 275 (39.5) | 0.71 (0.54, 0.93) |  |
| **Blood pressure** |  |  |  |  |  |
| <140/90 mmHg | - | 1,248 (70.8) | 1,525 (72.9) | 0.92 (0.79, 1.05) | 0.17 |
|  | + | 230 (62.2) | 484 (69.4) | 0.73 (0.56, 0.95) |  |
| <130/80 mmHg | - | 776 (44.0) | 856 (40.9) | 1.15 (1.01, 1.31) | 0.34 |
|  | + | 133 (36.0) | 254 (36.4) | 0.97 (0.74, 1.26) |  |
| **Lipids** |  |  |  |  |  |
| LDL-C** | - | 1,047 (59.4) | 1,373 (65.6) | 0.75 (0.66, 0.86) | 0.18 |
|  | + | 186 (50.3) | 427 (61.3) | 0.62 (0.48, 0.81) |  |
| Non-HDL-C*** | - | 1,160 (65.8) | 1,492 (71.3) | 0.77 (0.67, 0.88) | 0.70 |
|  | + | 222 (60.0) | 467 (67.0) | 0.71 (0.55, 0.93) |  |
| HDL-C | - | 1,679 (95.2) | 1,834 (87.6) | 2.85 (2.21, 3.68) | 0.79 |
| (≥1.03 mmol/L) | + | 342 (92.4) | 574 (82.4) | 2.62 (1.70, 4.05) |  |
| TG | - | 1,267 (79.1) | 1,378 (73.0) | 1.38 (1.18, 1.62) | 0.49 |
| (<1.68 mmol/L) | + | 252 (76.8) | 451 (72.2) | 1.20 (0.88, 1.65) |  |
| **Anthropometric indices** | |  |  |  |  |
| BMI | - | 1,161 (65.9) | 1,533 (73.2) | 0.67 (0.58, 0.77) | 0.25 |
| (<25 kg/m^2^) | + | 241 (65.1) | 476 (68.3) | 0.78 (0.60, 1.03) |  |
| WC (W: <80 cm) | - | 567 (32.2) | 1,522 (72.7) | 0.18 (0.15, 0.20) | 0.15 |
| (M: <90 cm) | + | 80 (21.6) | 461 (66.1) | 0.14 (0.10, 0.18) |  |
| **Lifestyle and psychological factors** | | |  |  |  |
| Non-current smoker | - | 1,640 (93.0) | 1,479 (70.7) | 5.72 (4.64, 7.06) | 0.15 |
|  | + | 347 (93.8) | 544 (78.1) | 3.95 (2.49, 6.27) |  |
| Dietary fiber intake | - | 110 (6.2) | 193 (9.2) | 0.63 (0.49, 0.80) | 0.83 |
| (≥20 g) | + | 21 (5.7) | 54 (7.8) | 0.66 (0.39, 1.12) |  |
| LTPA | - | 804 (45.6) | 1,186 (56.7) | 0.62 (0.54, 0.70) | 0.01 |
| (≥6.6 METs·h/w) | + | 138 (37.3) | 398 (57.1) | 0.43 (0.33, 0.56) |  |
| Sleep duration | - | 1,408 (79.9) | 1,793 (85.7) | 0.65 (0.55, 0.77) | 0.24 |
| (≥6 h) | + | 292 (78.9) | 609 (87.4) | 0.51 (0.36, 0.72) |  |
| Non-constipation | - | 1,246 (70.8) | 1,766 (84.5) | 0.45 (0.39, 0.53) | 0.01 |
|  | + | 218 (58.9) | 482 (69.3) | 0.68 (0.52, 0.89) |  |
| Depressive symptoms  (CES-D scale <16 points) | - | 1,587 (90.0) | 1,949 (93.1) | 0.64 (0.51, 0.81) | 0.72 |
| (CES-D scale <16 points) | + | 321 (86.8) | 635 (91.1) | 0.59 (0.39, 0.88) |  |

* Female/male odds ratios adjusted for age.

** LDL-C <2.59 mmol/L in participants with a history of coronary heart disease, <3.11 mmol/L in those without a history of coronary heart disease.

*** Non-HDL-C <3.37 mmol/L in participants with a history of coronary heart disease, <3.89 mmol/L in those without a history of coronary heart disease.

Triglycerides evaluated among participants with fasting values (n: women = 1,929, men = 2,512).

Six participants with missing values in defecation frequency were excluded from the analysis of constipation.

Abbreviations: BMI, body mass index; BP, blood pressure; CES-D, Center for Epidemiologic Studies Depression; CI, confidence interval; CVD, cardiovascular diseases; HDL-C, high-density lipoprotein cholesterol; Hx, history; LDL-C, low-density lipoprotein cholesterol; LTPA, leisure-time physical activity; M, men; MET h/w, metabolic equivalent hours per week; TG, triglycerides; W, women.

**Figure legends**

**Figure S1. Sensitivity analyses: achievement of recommended ranges for cardiovascular risk factors in men and women after multiple-adjustment.**

* Female/male odds ratios adjusted for age, duration of diabetes, body mass index, oral hypoglycemic agent and insulin use (for glycemic control), anti-hypertensive agent use (for BP control), and anti-hyperlipidemic agent use (for lipids control).

** LDL-C <2.59 mmol/L in participants with a history of coronary heart disease, <3.11 mmol/L in those without a history of coronary heart disease.

*** Non-HDL-C <3.37 mmol/L in participants with a history of coronary heart disease, <3.89 mmol/L in those without a history of coronary heart disease.

Triglycerides evaluated among participants with available data for fasting values (n: women = 1,778, men = 2,512).

Abbreviations: BP, blood pressure; CI, confidence interval; HDL-C, high-density lipoprotein cholesterol; LDL-C, low-density lipoprotein cholesterol; TG, triglycerides.

**Figure S2. Sensitivity analyses: achievement of recommended ranges for cardiovascular risk factors in men and women after excluding premenopausal participants.**

165 premenopausal participants were excluded from the sensitivity analyses.

* Female/male odds ratios adjusted for age.

** LDL-C <2.59 mmol/L in participants with a history of coronary heart disease, <3.11 mmol/L in those without a history of coronary heart disease.

*** Non-HDL-C <3.37 mmol/L in participants with a history of coronary heart disease, <3.89 mmol/L in those without a history of coronary heart disease.

Triglycerides evaluated among participants with available data for fasting values (n: women = 1,778, men = 2,512).

Six participants with missing values in defecation frequency were excluded from the analysis of constipation.

Abbreviations: BMI, body mass index; BP, blood pressure; CES-D, Center for Epidemiologic Studies Depression; CI, confidence interval; HDL-C, high-density lipoprotein cholesterol; LDL-C, low-density lipoprotein cholesterol; LTPA, leisure-time physical activity; TG, triglycerides.

**Figure S1**


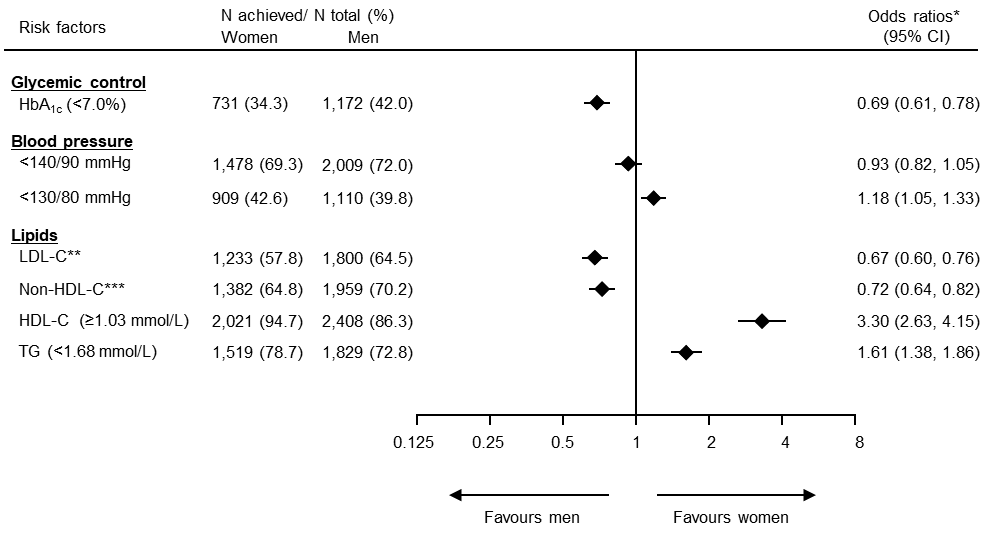


**Figure S2**


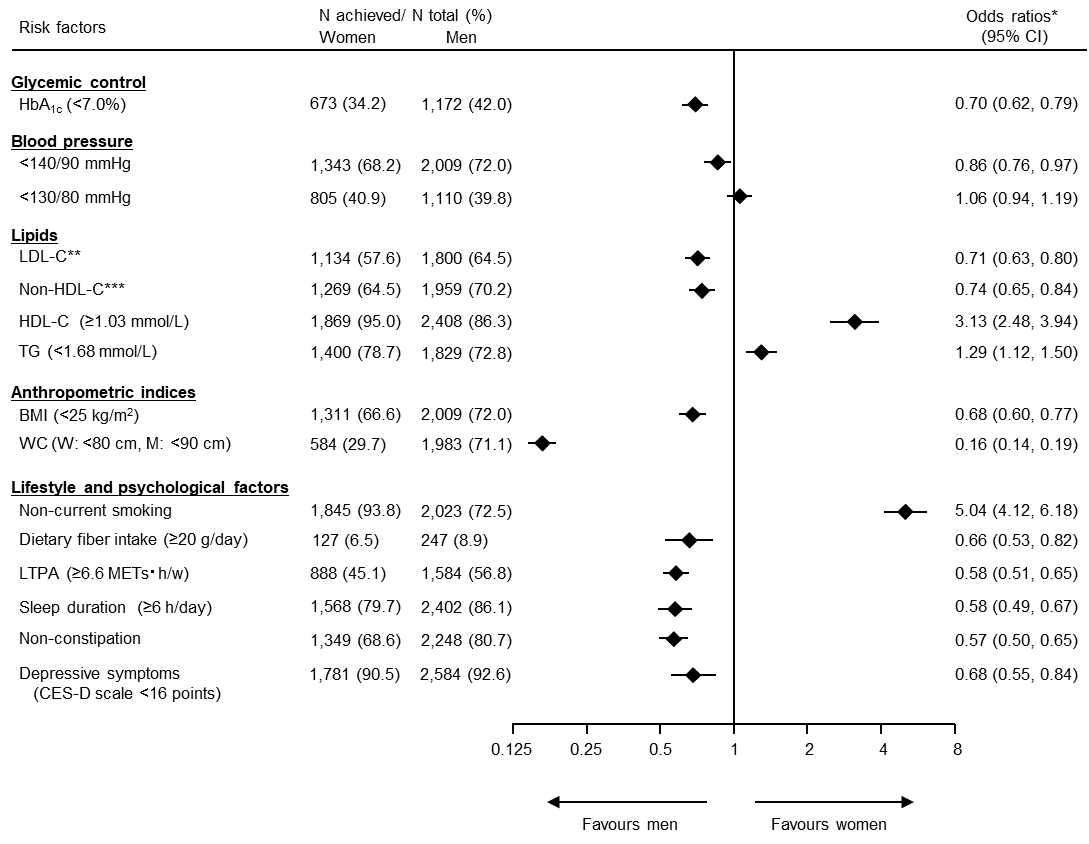

Supplement: Supplementary file 1 — Additional file 1: Table S1. Subgroup analyses: achievement of recommended ranges for cardiovascular risk factors in men and women according to age. Table S2. Subgroup analyses: achievement of recommended ranges for cardiovascular risk factors in men and women according to previous history of CVD. Figure S1. Sensitivity analyses: achievement of recommended ranges for cardiovascular risk factors in men and women after multiple-adjustment. Figure S2. Sensitivity analyses: achievement of recommended ranges for cardiovascular risk factors in men and women after excluding premenopausal participants. [file 13293_2023_517_MOESM1_ESM.docx]
